# Do Aging and Dual-Tasking Impair the Capacity to Store and Retrieve Visuospatial Information Needed to Guide Perturbation-Evoked Reach-To-Grasp Reactions? 

**DOI:** 10.1371/journal.pone.0079401

**Published:** 2013-11-05

**Authors:** Kenneth C. Cheng, Jay Pratt, Brian E. Maki

**Affiliations:** 1 Toronto Rehabilitation Institute, University Health Network, Toronto, Ontario, Canada; 2 Sunnybrook Health Sciences Centre, Toronto, Ontario, Canada; 3 Institute of Medical Science, University of Toronto, Toronto, Ontario, Canada; 4 Department of Psychology, University of Toronto, Toronto, Ontario, Canada; 5 Institute of Biomaterials and Biomedical Engineering, University of Toronto, Toronto, Ontario, Canada; 6 Department of Surgery, University of Toronto, Toronto, Ontario, Canada; University of Tokyo, Japan

## Abstract

A recent study involving young adults showed that rapid perturbation-evoked reach-to-grasp balance-recovery reactions can be guided successfully with visuospatial-information (VSI) retained in memory despite: 1) a reduction in endpoint accuracy due to recall-delay (time between visual occlusion and perturbation-onset, PO) and 2) slowing of the reaction when performing a concurrent cognitive task during the recall-delay interval. The present study aimed to determine whether this capacity is compromised by effects of aging. Ten healthy older adults were tested with the previous protocol and compared with the previously-tested young adults. Reactions to recover balance by grasping a small handhold were evoked by unpredictable antero-posterior platform-translation (barriers deterred stepping reactions), while using liquid-crystal goggles to occlude vision post-PO and for varying recall-delay times (0-10s) prior to PO (the handhold was moved unpredictably to one of four locations 2s prior to vision-occlusion). Subjects also performed a spatial- or non-spatial-memory cognitive task during the delay-time in a subset of trials. Results showed that older adults had slower reactions than the young across all experimental conditions. Both age groups showed similar reduction in medio-lateral end-point accuracy when recall-delay was longest (10s), but differed in the effect of recall delay on vertical hand elevation. For both age groups, engaging in either the non-spatial or spatial-memory task had similar (slowing) effects on the arm reactions; however, the older adults also showed a dual-task interference effect (poorer cognitive-task performance) that was specific to the spatial-memory task. This provides new evidence that spatial working memory plays a role in the control of perturbation-evoked balance-recovery reactions. The delays in completing the reaction that occurred when performing either cognitive task suggest that such dual-task situations in daily life could increase risk of falling in seniors, particularly when combined with the general age-related slowing that was observed across all experimental conditions.

## Introduction

Rapid reach-to-grasp reactions are often executed to prevent falling in response to a sudden “loss of balance” perturbation, and such reactions are particularly important for older adults [1,2]. Recent studies of perturbation-evoked reactions have indicated that both young and older adults can perform functionally-adequate reach-to-grasp reactions without concurrent visual fixation of the handrail [3,4], and can execute these reactions even when vision is completely occluded following balance perturbation-onset (PO) [5-7]. This has led to speculation that these reactions may be guided, in daily life, by visuospatial information (VSI) that is acquired and stored proactively (prior to PO) through natural exploratory gaze behaviour [3-5]. Such a strategy avoids the delay that would occur if it was necessary to acquire and process online visual information about potential handhold locations after the onset of a sudden unexpected balance perturbation. 

The ability to use VSI stored in visuospatial memory to guide rapid reach-to-grasp reactions is further demonstrated by a study where additional challenges were imposed by: 1) substantially prolonging the length of time that VSI about the handhold location had to be retained in visuospatial memory prior to PO (i.e. “recall-delay” of up to 10s), and 2) performing a concurrent cognitive task during the recall-delay interval [8]. These task conditions were intended to simulate the daily-life situation where potential handhold locations are mapped upon first entering an environment, but the balance perturbation occurs several seconds later. Results showed that healthy young adults were always able to recover balance successfully by reaching to grasp a small handhold that changed position unpredictably prior to visual occlusion, even though recall-delay did lead to some reduction in endpoint accuracy and the additional cognitive task did lead to some slowing of the reactions [8]. 

The capacity of older adults to execute effective reach-to-grasp reactions under such task conditions has not been established, but there are several reasons why this could be impaired. In particular, age-related declines in attention [9], and in the encoding [10] and retention [11,12] of visuospatial memory [13] could adversely affect the capacity to retain accurate VSI about handhold location for a prolonged time interval prior to PO. Furthermore, the ability of older adults to retain and retrieve stored-VSI could be further compromised if required to perform a concurrent cognitive task during that time interval. This situation is especially pertinent to everyday conditions where balance disturbances often occur while cognitive attention is being shared by other motor or non-motor tasks [14]. 

The objective of the present study was to determine the effects of recall-delay and concurrent cognitive-task performance on the ability of healthy older adults to use stored-VSI to guide effective reach-to-grasp balance-recovery reactions. Liquid-crystal goggles were used to force reliance on stored-VSI by occluding vision immediately after PO. To vary the length of time that handhold location had to be retained in memory, vision was also occluded for varying recall-delay times (0-10s) immediately prior to PO. In a subset of trials, subjects performed a concurrent spatial or non-spatial memory task during the recall-delay interval. Results were compared to a previous study in which healthy young adults completed the same protocol [8]. 

We hypothesized that the recall-delay effects previously observed in young adults (i.e. reduced reach accuracy [8]) would be exacerbated in older adults, and that these effects would be accompanied by an increased frequency of overt motor errors (e.g. hand-handhold collision and incomplete grasp formation). Conversely, we hypothesized that aging would not exacerbate the effects of cognitive-task performance previously observed in young adults (i.e. slowing of the reactions [8]), in view of evidence that older adults tend to prioritize postural control [15-17]. Instead, we hypothesized that the older adults would exhibit a dual-task cost in the performance of the spatial-memory task [18,19].

## Methods

### Participants

Ten naïve, community-dwelling, healthy older adult subjects [four male, six female; ages 60-76 years (mean=70); mass 50-88kg (mean=70); height 150-182cm (mean=164)] were tested and results were compared to ten younger-adult subjects tested in the previous study [five male, five female; ages 22-30 years (mean=26); mass 47-98kg (mean=68); height 155-184cm (mean=169)] [8]. All subjects were right-handed, without a recent history of falling, and able to stand and walk without aid. Exclusion criteria included diabetes, neurological or sensory disorders, recurrent dizziness or unsteadiness, use of medications that may affect balance, joint replacement, medical conditions interfering significantly with daily activities, or functional limitations of limb use. Subjects were required to have a minimum Mini-Mental-Status-Examination score of 24/30 and a minimum Snellen visual acuity of 20/40 without spectacles. As indicated in Table 1, the older adults had some notable age-related changes in their visual and cognitive function. The experimental protocol described in the article was approved by the Ethics Review Board of the Sunnybrook Health Sciences Centre, in accordance with the Helsinki declaration. Each subject provided written informed consent to comply with ethics approval granted by the institutional review board.

**Table 1:** Visual and cognitive function.

	**Mean ± SD** [**min, max**] ^A^		**Comparative data from other older-adult studies ^B^**			
	***Young adults***	***Older adults***	***Mean ± SD***	***n***	***Age***	***References***
***Visual acuity:***						
Snellen acuity score ^C^	1.2 ± 0.3 [0.8, 1.5]	0.7 ± 0.2 [0.5, 1.0] **	0.55 ^D^	54	75 ^D^	[76]
***Contrast sensitivity:***						
Melbourne Edge Test (dB) ^E^	23.5 ± 0.5 [23, 24]	22.2 ± 1.6 [19, 24] *	20.3 ± 2.6	156	77 ± 5	[77]
***Depth perception:***						
Howard-Dolman test (cm) ^F^	-0.3 ± 0.4 [-1.0, 0.3]	0.0 ± 1.7 [-2.6, 3.1]	2.7 ± 4.6	156	77 ± 5	[77]
***Useful field of view:***						
Subtest 1: Processing speed (ms)	17.6 ± 1.3 [17, 20]	17.3 ± 1.0 [17, 20]	31 ± 41	2759	74 ± 6	[78]
***Simple reaction time:***						
Hand reaction time (ms)	204 ± 19 [181, 234]	244 ± 38 [204, 317] **	281 ± 58	156	77 ± 5	[77]
***Spatial working memory:***						
Brooks’ spatial letter task (error %)	0.2 ± 0.6 [0, 2]	12.9 ± 7.6 [4, 26] **	9.72 ^G^	15	70 ± 6	[15]
***Cognitive function:***						
Mini-Mental-Status-Examination	Not tested	27.0 ± 1.2 [25.5, 29]	26.1 ± 3.1	406	79 ± 6	[79]
Trail making test A (s) ^H^	17 ± 5 [10, 25]	46 ± 20 [22, 78] **	42.6 ± 15.3	311	72 ± 4	[80]
Trail making test B (s) ^H^	41 ± 12 [21, 55]	117 ± 74 [42, 267] **	103.3 ± 40.4	311	72 ± 4	[80]

**Note:**

^A^ Significant differences (Student’s unpaired t-test) between the two age-groups were indicated by * (p<0.05) and ** (p<0.01). Smaller scores indicate better test-performance for all measures except for Snellen acuity test, Melbourne Edge Test and Mini-Mental-Status-Examination

^B^ All studies involved healthy community-dwelling older adults, except Lord and Menz (2000) used a combination of community-dwelling (n=77) and nursing-home residents (n=79)

^C^ Expressed in Snellen decimal, for example, 20/40 = 0.5

^D^ Standard deviation was not reported

^E^ dB: decibels (ranging from 1=poor vision to 24=good vision)

^F^ Error in matching positioning of rods

^G^ Determined during dual-task balance testing; standard deviation was not reported

^H^ Trail making test A measures cognitive processing speed, while Trail making test B measures executive functioning

### Protocol

**Figure 1:**
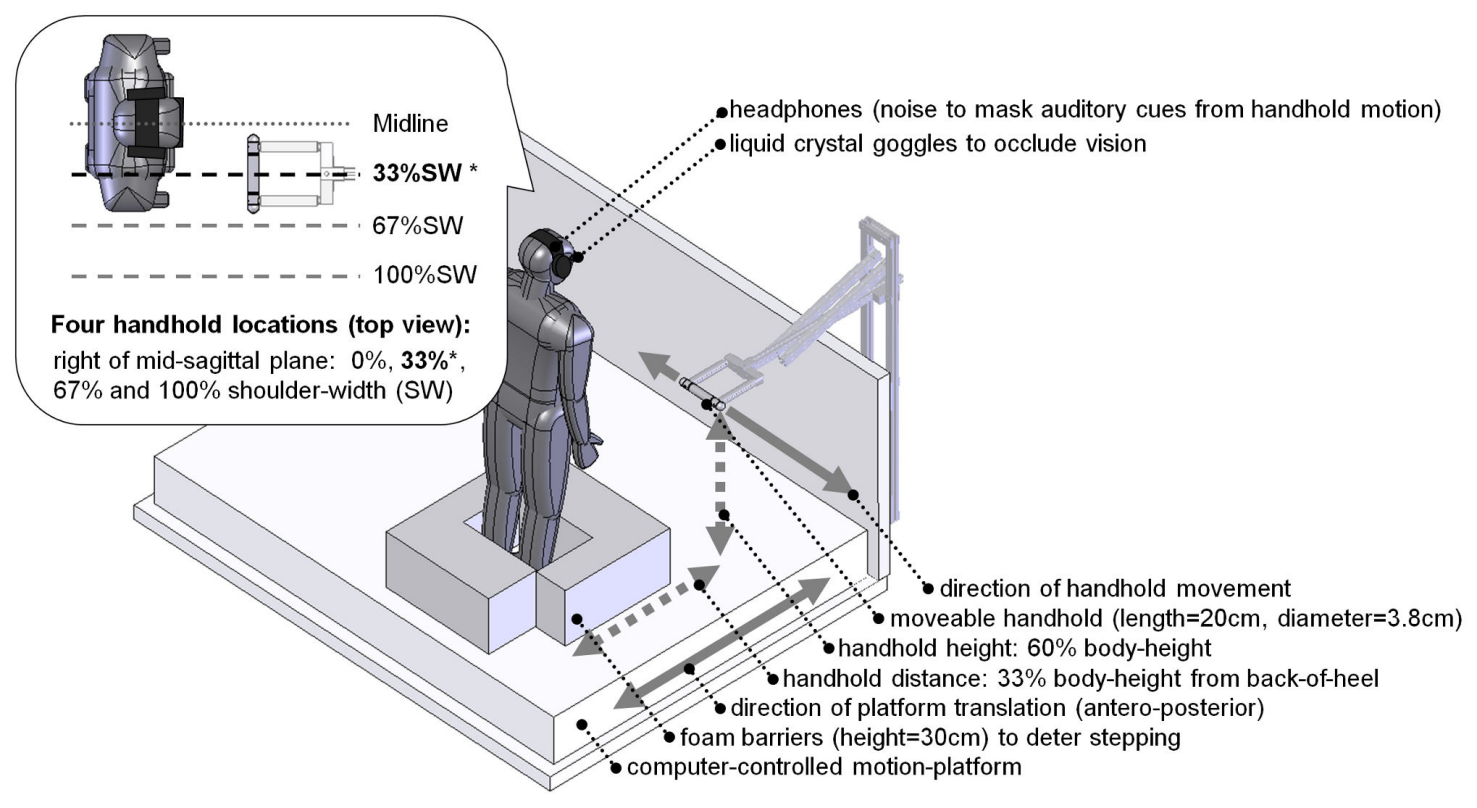
Motion platform and moveable handhold systems. Dashed lines in the insert indicate the four handhold positions that were tested. Analysis focused on the highlighted handhold position (33% shoulder-width*) and anterior platform translations (backward falling motion).

The protocol was identical to an earlier study involving only healthy young adults [8]. Balance-recovery reach-to-grasp reactions were evoked by sudden forward (0.12m, 0.41m/s, 1.4m/s^2^) or backward (0.18m, 0.6m/s, 2.0m/s^2^) translation of a 2m×2m computer-controlled motion-platform [20]. In each trial, a motor-driven device [21] mounted on the platform controlled a cylindrical handhold to move along a transverse axis in front of the subject (distance from handhold to back of heels=33% of body height; handhold height=60% of body height) and to stop unpredictably at one of four locations [0%, 33%, 67% or 100% of shoulder-width (SW) to the right of the mid-sagittal plane; Figure 1]. The focus of the study was on the grasping reactions evoked by forward platform translation (backward falling motion), in trials where the handhold was positioned at 33% of shoulder-width from the mid-sagittal plane; however, other combinations of handhold position and perturbation direction were also included to increase unpredictability and deter anticipatory reactions or other proactive strategies (see Table 2). For safety, subjects wore a harness designed to prevent impact between body and floor, as well as padded gloves and wrist guards to reduce impact to the hands and wrist if and when collision to the back of hand or wrist occurred. The padding was only on the dorsal side of the hand and did not interfere with grasping.

**Table 2:** Distribution of trials (tested in random order).

**Recall delay time**	**No secondary task**	**Non-spatial-memory task**	**Spatial-memory task**
	**3focustrials**		
**0s**	2 other fwd-translation trials	0 trials *	0 trials *
	3 bwd-translation trials		
	**3focustrials**		
**2s**	2 other fwd-translation trials	0 trials *	0 trials *
	3 bwd-translation trials		
	**3focustrials**	**3focustrials**	**3focustrials**
**5s**	2 other fwd-translation trials	2 other fwd-translation trials	2 other fwd-translation trials
	3 bwd-translation trials	3 bwd-translation trials	3 bwd-translation trials
	**3focustrials**	**3focustrials**	**3focustrials**
**10s**	2 other fwd-translation trials	2 other fwd-translation trials	2 other fwd-translation trials
	3 bwd-translation trials	3 bwd-translation trials	3 bwd-translation trials
	4 catch trials	4 catch trials	4 catch trials

**Note: “Focus trials” = trials included in the analyses:**

- forward platform translation (backward falling motion); handhold at 33% shoulder-width from mid-line

**Additional trials included to increase unpredictability**:

- “other fwd-translation trials” = forward platform translation (backward falling motion); handhold at one of the three other handhold positions (see Figure 1 inset)

- “bwd-translation trials” = backward platform translation (forward falling motion); handhold at any of the four handhold positions

- “catch trials” = no platform motion (to deter anticipatory reactions); handhold at any of the four handhold positions

* Recall-delay interval not long enough to permit the secondary cognitive tasks to be performed.

**Figure 2:**
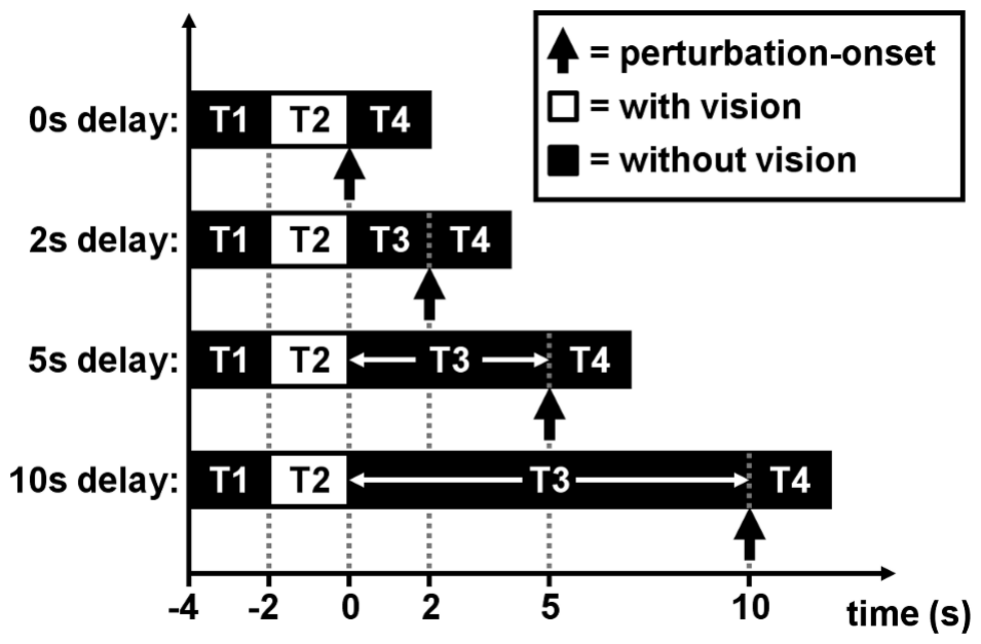
Sequence and timing of events. Liquid-crystal goggles occluded vision for 2s (interval T1) at the start of each trial, while the movable handhold moved to and stopped at one of four possible locations. Vision was then allowed for 2s (T2) and subjects were instructed to look at the handhold during this viewing period. Vision was then occluded again, for a recall-delay interval of 0s, 2s, 5s or 10s (T3) prior to perturbation-onset, and continued to be occluded for 2s after PO (T4), i.e. until the end of trial. For trials with recall delays of 5s or 10s, subjects performed either a spatial-memory task, a non-spatial-memory task or no secondary task during the recall-delay interval (T3).

Custom-modified [22] translucent liquid-crystal goggles (Translucent Technologies Inc., Toronto, ON) were used to occlude vision during a portion of each trial. At the start of each trial, vision was first occluded for 2s (interval T1 in Figure 2) while the movable handhold moved to and stopped at one of the four handhold positions. Vision was then allowed for 2s (interval T2), during which subjects were instructed to look at and remember the handhold location without turning their head. Vision was then occluded again, for a recall-delay interval (T3) of 0s, 2s, 5s or 10s prior to perturbation-onset, and continued to be occluded until the end of the trial (i.e. 2s after perturbation-onset). 

**Figure 3:**
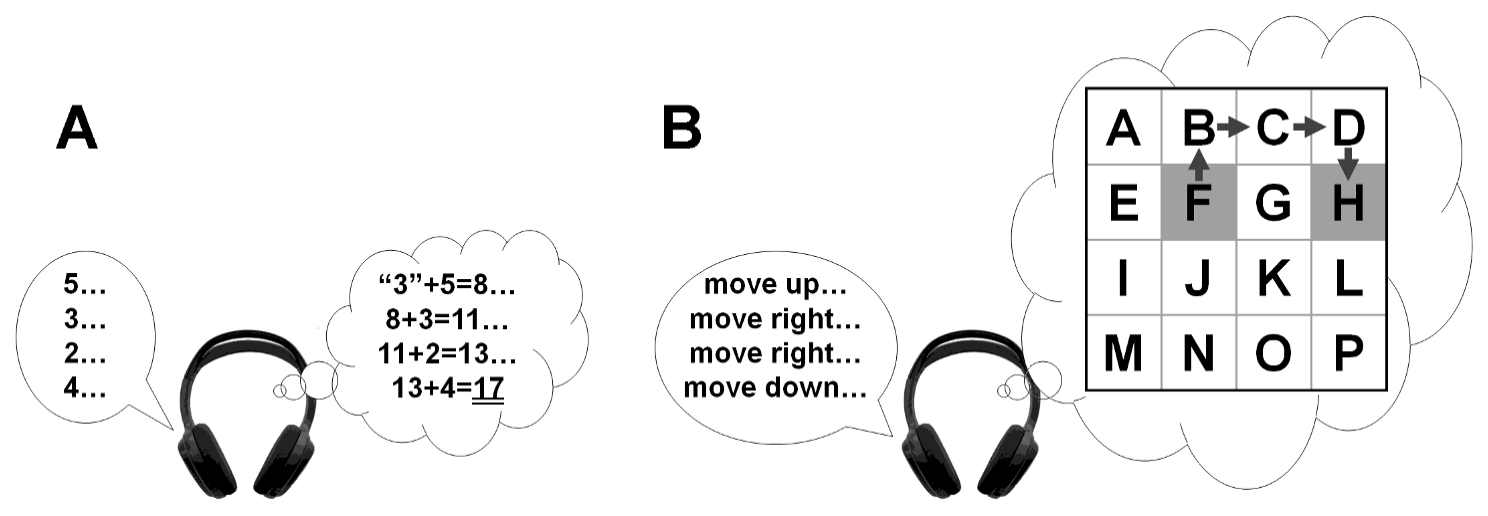
Cognitive tasks. (A) For the non-spatial-memory task, subjects were asked to add a series of auditorily-presented random numbers and to report the final sum after the end of the trial. Difficulty of the task was adjusted by changing the range of numbers (i.e. 1-to-3, 1-to-5, 1-to-9, or 4-to-12) to be added. Starting at the number “3”, subjects were instructed to sequentially add a series of random numbers delivered (via headphones) every 1.25s. As an example, the figure shows the correct response (3+5+3+2+4=17) to the sequence of numbers shown (2–5). (B) For the spatial-memory task, subjects were instructed to imagine a highlighted square moving around in an N×N matrix and to report the final position of the highlighted square within the matrix. Difficulty of the task was adjusted by changing the size of the matrix (i.e. 3×3, 4×4, 5×5, 6×6, or 7×7). Starting at or near the center cell, a random verbal command to move up, down, left or right was given every 1.25s. As an example, the gray arrows in the figure show the correct responses to a sequence of commands to “move up,” “move right,” “move right,” and “move down,” after starting at cell “F”. Subjects were shown the matrix after each trial and asked to identify the correct final response (cell “H” in the example shown).

For the trials with recall-delays of 5s and 10s, subjects performed a spatial-memory task (modified Brooks spatial task) or a non-spatial memory (mental arithmetic) task during the recall-delay interval, or performed no cognitive task at all (see 8 and Figure 3A and 3B for details). Note that the secondary tasks could not be performed in the absence of any recall-delay interval (i.e. in the trials where recall-delay time = 0s) and were also not included in the trials with a 2s recall-delay time because the 2s interval would have permitted only one mental computation (since each auditory number/instruction was given every 1.25s) and hence would not have allowed meaningful assessment of cognitive-task performance. 

All subjects practiced the cognitive tasks for 20-30min prior to balance-perturbation testing, while sitting in front of a computer. We then performed a series of trials to determine the cognitive task conditions to be used for each individual subject during the balance-perturbation testing. Our objective was to match difficulty level (within and across subjects) such that the tasks were challenging for each individual, but were not so difficult that subjects would “give up.” To this end, our goal was to adjust the task difficulty so that the correct “answer” was reported in ~70-90% of trials. Starting with the easiest task difficulty (adding numbers ranging from 1-to-3 in the non-spatial-memory task, or imagining a 3×3 array in the spatial-memory task), each subject performed blocks of five trials. For each new trial block, task difficulty was increased by one increment if the subject attained a minimum accuracy of 60% (3-out-of-5 correct responses) in the previous trial block, or decreased by one increment if the subject failed to achieve this cut-off rate. Task difficulty was chosen after the same difficulty level was failed twice. Additional trials were then performed to establish baseline (single-task) error-rates for the purposes of the dual-task analysis (five trials for the 5s task duration and five trials for the 10s duration); see Table 3.

**Table 3:** Cognitive-task performance.

	**Non-spatial-memory task**				**Spatial-memory task**			
**Subject**	**difficulty ^†^**	**single ^¶^**	**dual ^¶^**	**Δ error%^§^**	**difficulty ^†^**	**single ^¶^**	**dual ^¶^**	**Δ error% ^§^**
young 01	1-to-5	2/10	8/16	30%	6×6	2/10	3/16	-1%
young 02	1-to-5	3/10	2/16	-18%	4×4	2/10	1/16	-14%
young 03	1-to-5	0/10	0/16	0%	6×6	1/10	2/16	3%
young 04	1-to-5	1/10	4/16	15%	6×6	2/10	3/16	-1%
young 05	1-to-5	3/10	1/16	-24%	7×7	2/10	4/16	5%
young 06	4-to-12	2/10	4/16	5%	6×6	2/10	2/16	-8%
young 07	1-to-5	3/10	4/16	-5%	6×6	1/10	5/16	21%
young 08	1-to-5	1/10	2/16	3%	6×6	0/10	3/16	19%
young 09	1-to-3	1/10	3/16	9%	6×6	1/10	3/16	9%
young 10	1-to-9	2/10	5/16	11%	6×6	1/10	3/16	9%
older 01	1-to-9	3/10	7/16	14%	6×6	2/10	14/16	68%
older 02	1-to-3	3/10	8/16	20%	4×4	2/10	14/16	68%
older 03	1-to-5	2/10	1/16	-14%	5×5	3/10	12/16	45%
older 04	1-to-3	1/10	2/16	3%	5×5	1/10	9/16	46%
older 05	1-to-9	1/10	1/16	-4%	6×6	2/10	8/16	30%
older 06	1-to-5	2/10	4/16	5%	5×5	3/10	9/16	26%
older 07	1-to-5	2/10	2/16	-8%	4×4	2/10	7/16	24%
older 08	1-to-5	1/10	6/16	28%	5×5	3/10	6/16	8%
older 09	1-to-5	3/10	10/16	33%	4×4	3/10	9/16	26%
older 10	1-to-5	2/10	7/16	24%	4×4	3/10	9/16	26%

**Note:** ^†^ Non-spatial task difficulty was adjusted by changing the range of possible numbers to be summed (see Figure 3A); spatial-memory task difficulty was adjusted by changing the N×N matrix size (see Figure 3B).

^¶^ Proportion of trials with error during single-task trials (no balance perturbations) and dual-task trials. Single-task error rates were based on five 5s trials and five 10s trials; dual-task error rates were based on eight trials with a 5s recall-delay interval and eight trials with a 10s recall-delay interval.

^§^ Difference in percentage error rate (dual-task error rate minus single-task error rate).

During the main (balance-perturbation) experiment, subjects were informed of the cognitive task at the start of each trial, and were instructed to face forward, with arms resting at sides and the hands forming a relaxed fist with thumb “on top.” To recover balance, they were further instructed to grasp a marked (with red tape) “target” section of the handhold (125% of hand-width) as quickly as possible after the onset of the platform motion, without moving their feet (deterred with 30cm-high foam-rubber barriers). Additional motivation to produce rapid reach-to-grasp responses was provided in the form of a $50 prize, awarded to the subject who achieved the quickest average response time. Subjects were told that premature initiation of arm movements (prior to onset of platform movement) or errors in performing the cognitive task would result in a penalty that reduced their chances of winning the $50 prize. To minimize mental fatigue and adaptive effects, the order of testing the trials was completely randomized for each subject. To further reduce adaptive effects, ten practice perturbation trials were performed prior to the start of the experiment (three spatial-task trials, three non-spatial-task trials and four no-cognitive-task trials).

### Data collection and analysis

Video recordings from four cameras were used to determine which arm was used to grasp the handhold, whether a full grasp was achieved (all digits wrapped around the handhold), whether a collision error occurred (contact with wrist, or back of hand or digits), and whether the subject attempted to step (by kicking the foam-rubber barriers) or fell into the safety harness (confirmed by load cell on harness cable). Surface electrodes were used to record electromyographic (EMG) activity bilaterally in the anterior deltoid, lateral deltoid and biceps muscles (band-pass filtered, 10-500Hz; sampling-rate=1000Hz). Reaction time was defined as the earliest EMG onset latency in any of these muscles (in the reaching arm), as determined by a computer algorithm [23] and confirmed by visual inspection. Contact time was detected by force-sensing resistors mounted on the front, back and top of the handhold (sampling-rate=200Hz), and confirmed using a hand-velocity criterion (<5% peak resultant velocity), as determined by the motion-analysis system described below. Movement time was defined as the difference between contact- and reaction-time. Reaction- and contact-time were defined relative to onset of platform acceleration (>0.1m/s^2^), determined by an accelerometer mounted on the motion platform.

A three-dimensional motion-analysis system (Vicon-Peak Performance; Englewood, CO) collected kinematic data [sampling rate=200Hz; displacement data low-pass filtered at 6Hz [24] using a dual-pass fourth-order Butterworth filter (99% of marker signal power was found to be <6Hz in unfiltered trials)]. Analysis focused on the location and trajectory of the right hand (marker on third-metacarpal knuckle), relative to markers mounted at each end of the handhold. The hand-marker data were used to determine the maximum resultant hand velocity in the transverse plane, time-to-peak-velocity [relative to the movement-onset time (vertical hand velocity >5% of peak)], and time-after-peak-velocity (time from peak velocity to handhold contact). The location of the hand marker relative to the center of the handhold target area, at time of handhold contact, was used to define the reach error in each coordinate direction. 

Data from the hand marker were also used to describe the trajectory of the reach in the transverse plane. Trajectory data were used to determine the deviation from the ‘direct-path’ to the handhold [25,26], as well as the maximum lateral direct-path deviation and maximum vertical hand elevation [8]. The direct-path was defined as the straight-line path connecting the hand position at movement onset to the center of the target region of the handhold. Orthogonal deviation from this direct-path was calculated at increments of 5% of the direct-path distance.

Repeated-measures analysis of variance (ANOVA) and post-hoc Tukey multiple comparisons were performed to test the hypotheses (criterion level of significance: 0.05). The primary dependent variables were: 1) reach timing (reaction-onset, movement-time and contact-time); 2) reach velocity (peak velocity, time-to-peak-velocity and time-after-peak-velocity); 3) reach accuracy and variability; 4) grasp formation (frequency of full-grasp and frequency of hand-handhold collision errors); and 5) cognitive-task error rate (see Table 4 for summary). Other variables analyzed included: 1) orthogonal deviation from a direct-path trajectory in transverse plane; and 2) maximum vertical elevation and lateral deviation of the hand during the trajectory. The reach accuracy and variability variables were analyzed separately in each coordinate direction, and were expressed as a proportion of subject height prior to analysis (to control for variation related to differences in body size). All data were rank-transformed prior to analysis to avoid errors arising from violations of the assumptions (i.e. normality of residuals and homogeneity of variance) underlying the ANOVA [27-29]. However, in view of evidence that analysis of rank-transformed data may not provide a robust test for interactions [30], we also performed the analyses on the untransformed data and compared the interaction findings. 

**Table 4:** Summary of variables.

**Variables and categories**	**Descriptions**
***Reach timing:***	Reach-to-grasp reactions have to be completed rapidly to
Reaction time	prevent a fall (i.e. fast contact time). This is achieved via
Movement time	early initiation (reaction time) and/or fast execution
Contact time	(movement time) of the reaching movement.
***Reach velocity:***	Rapid reach velocity reduces movement time. Shorter
Peak velocity	time-to-peak-velocity suggests faster acceleration of the
Time-to-peak-velocity	reaching hand, whereas longer time-after-peak-velocity
Time-after-peak-velocity	allows more time for correction of the reach trajectory.
***Reach trajectory:***	Transporting the hand along a shorter path-of-travel (i.e.
Medio-lateral deviation from the “direct-path”	with less deviation from the direct (straight-line path) in
Maximum vertical hand elevation	the horizontal plane and with lower elevation in the
Maximum lateral deviation from the “direct-path”	vertical plane) is more efficient but requires more
	accurate mapping of the final target position.
***Reach accuracy:***	Reach accuracy is important especially if the graspable
Hand contact position in all three axes	object is small. Both systematic and variable errors
Variability of hand contact position in all three axes	reflect the accuracy with which the target was encoded,
	stored and retrieved from working memory.
***Prehension (grasp formation):***	Accurate prehension (i.e. achieving a full grasp without
Frequency of full grasp	colliding with the handhold) provides a stable support
Frequency of hand-handhold collision	and anchor to restore postural equilibrium.
***Cognitive-task performance:***	Impaired cognitive-task performance during the balance
Cognitive-task error rate (dual-task trials relative to	task suggests that both tasks competed for the same
single-task trials)	cognitive resources, and that the combined demands of
	the two tasks exceeded the available capacity.

As noted above, the cognitive tasks could only be performed in trials having a sufficiently long recall-delay interval, i.e. 5s or 10s. Since the shorter recall-delay times (0s and 2s) could only be tested in trials having no secondary task, the no-secondary-task trials were used to determine the main effects due to recall-delay time (DT = 0s, 2s, 5s, or 10s) and the extent to which these effects differed in the young and older adults (age×recall-delay interaction) (i.e. two-way ANOVA, factors = recall-delay time and age group). Since the cognitive tasks could only be performed in the trials with 5s or 10s recall-delay times, these trials were used to determine the main effects due to cognitive task (no task, spatial task, or non-spatial task) and the extent to which these effects differed in the young and older adults (age×cognitive-task interaction). To account for variance due to recall delay, recall-delay time (DT = 5s or 10s) and associated interactions were also included in these latter analyses, in addition to cognitive task and age group (i.e. three-way ANOVA). 

For the frequency variables, the proportion of trials in which the event was observed was calculated within each subject, for each of the experimental conditions, and the ANOVA was performed on the rank-transformed proportions. To analyze accuracy variability, the standard deviation of the grasp error was determined within each subject, for each experimental condition, and the ANOVA was performed on the rank-transformed standard deviations. To analyze deterioration in cognitive-task performance during the balance-perturbation trials, the percentage of trials with incorrect responses was determined within each experimental condition, for each subject, and subtracted from the subject’s baseline (single-task) incorrect-response rate; the ANOVA was performed on the rank-transformed difference in rates. For all of the other variables, rank-transformed data from individual trials were used. As noted earlier, the protocol was designed to focus on trials involving forward platform translation, with the handhold located at 33% of shoulder-width from the mid-sagittal plane. Each subject performed 24 such “focus trials" (see Table 2); therefore, there were a total of 480 trials available for analysis.

## Results

### Overview

Like the previously-tested younger subjects [8], the older adults in this study were generally able to perform functionally-adequate reach-to-grasp reactions to restore postural stability, despite having to “remember” the handhold location for up to 10s, even when required to perform a concurrent spatial or non-spatial cognitive task. Subjects never fell into the safety harness (maximum harness loading was below 5% of body weight in all trials) nor stepped to recover their balance (i.e. by kicking or stepping over the foam barriers), and there were only two trials (in two older adults) where the initial reaching movement missed the handhold. 

Detailed descriptions of the results of the statistical analyses are provided below, and a listing and description of the analyzed variables are provided in Table 4. Briefly, the main findings were as follows:

1. The older adults tended to have slower reach-to-grasp responses than the young adults, across all experimental conditions, but there was limited evidence that aging led to less accurate reaching or less effective grasping.
2. The length of time that the handhold location had to be retained in memory (recall-delay time) had relatively little effect on the timing and speed of the reach-to-grasp reactions; however, there was a reduction in medio-lateral reach accuracy when required to retain the handhold location in memory for the longest time interval (recall-delay of 10s), and this was accompanied by a tendency for greater lateral excursion of the hand to occur as it moved toward the handhold. Generally, similar effects were seen in both age groups; however, there was one difference: the older adults raised their hand to a similar height for all recall delays, whereas the young adults lifted their hand less high during the shorter-delay trials.
3. Performing a secondary cognitive task during the recall-delay interval led to slowing of the reach-to-grasp reactions, with similar effects in both age groups. Cognitive-task errors occurred more frequently in this dual-task situation, in comparison to the baseline single-task trials performed while sitting quietly. Moreover, the ability to perform the cognitive task accurately in the dual-task situation deteriorated to a greater extent in the older adults than in the young adults, and particularly so when performing the spatial, rather than non-spatial, cognitive task.

### Main effects due to age

**Figure 4:**
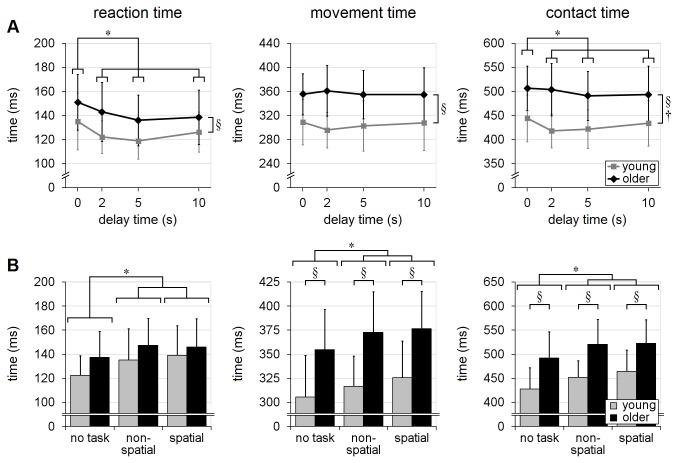
Timing variables. Effects of recall-delay and cognitive-task on reach-to-grasp timing (EMG-onset, movement and handhold-contact times) are shown in (A) and (B), respectively. Means and standard deviations are shown for young adults (gray) and older adults (black). Note the slower reaction-time and contact-time during no-delay trials in (A), as well as the slower timing during dual-task trials in (B). Significant age×recall-delay interaction (†) in contact-time in (A) suggested that older adults had relatively consistent contact time throughout all recall-delay conditions in comparison to young adults. § indicates a significant difference due to age (main effect); * indicates a significant difference due to recall-delay in (A) or cognitive-task in (B) (α=0.05); whiskers indicate standard deviations.

**Figure 5:**
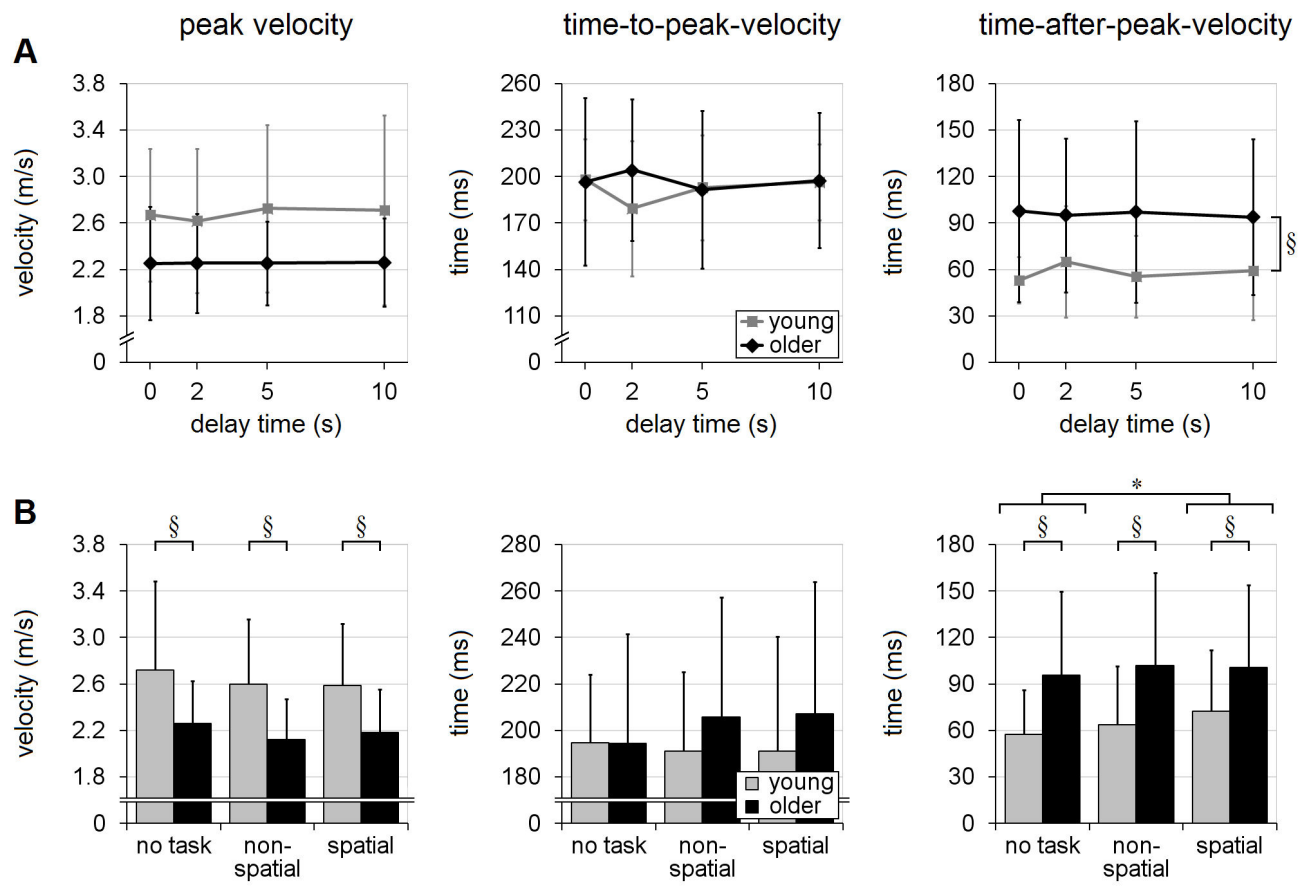
Hand-velocity variables. Effects of recall-delay and cognitive-task on hand velocity (peak resultant velocity in the transverse plane, time-to-peak-velocity and time-after-peak-velocity) are shown in (A) and (B), respectively. Means and standard deviations are shown for young adults (gray) and older adults (black). Note that older adults tended to achieve lower peak velocity, as well as spending significantly longer time on deceleration (i.e. larger time-after-peak-velocity). § indicates a significant difference due to age (main effect); * indicates a significant difference due to cognitive-task in (B) (α=0.05); whiskers indicate standard deviations.

**Figure 6:**
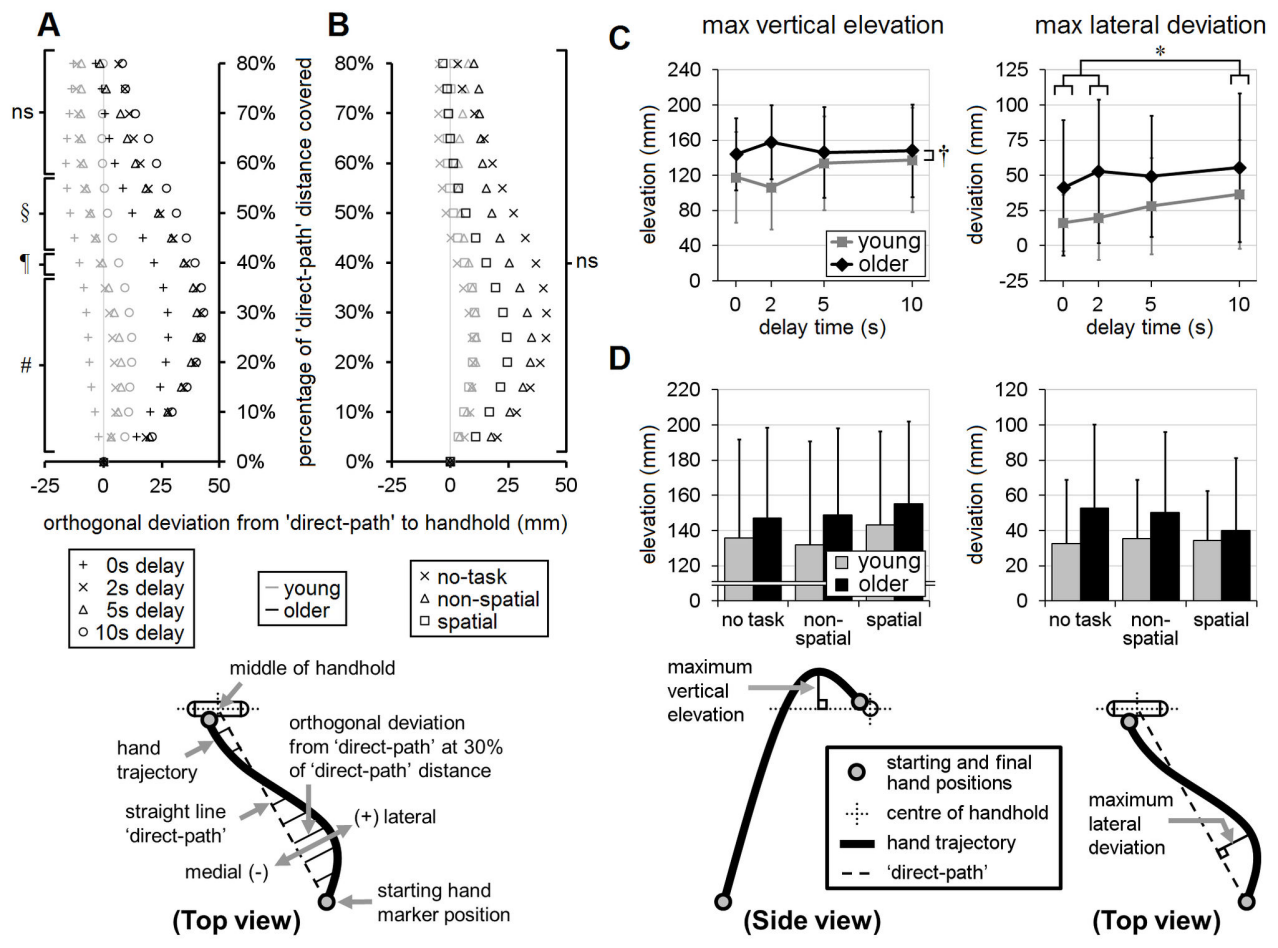
Hand trajectory and maximum hand displacement. Effects of recall-delay and cognitive-task on mean hand trajectory (A and B, respectively) and on maximum hand displacement (C and D, respectively) are shown in these figures. Means and standard deviations are shown for young adults (gray) and older adults (black); whiskers in panels (C) and (D) indicate standard deviations. The trajectories and maximum lateral hand displacement are defined relative to the “direct-path” connecting the starting hand position and the center of the handhold in the transverse plane (see inset schematic drawings). Panels (A) and (B) illustrate: (1) the tendency of older adults to transport the hand along a more curvilinear and lateral trajectory; (2) the tendency (in both age groups) for lateral hand excursion to increase with increasing recall-delay time (DT); and (3) the lack of any significant cognitive-task effect on hand trajectory [DT=0s significantly different from DT=2-10s (#); DT=0s significantly different from DT=5-10s (¶); DT=0s significantly different from DT=10s (§); no significant differences between means (ns)]. Panel (C) illustrates: (1) a significant effect of recall-delay (*) on maximum lateral hand deviation (further supporting the trends seen in the trajectory plots); and (2) a significant age×recall-delay interaction (†) in maximum vertical hand elevation (suggesting a more consistent tendency for older adults to raise the hand equally high across all recall-delay conditions). [Note: the data were expressed as a proportion of body height in the analyses to reduce variation related to differences in body size; however, the data are shown here in mm to make it easier to interpret the magnitude of the differences in hand displacement].

**Figure 7:**
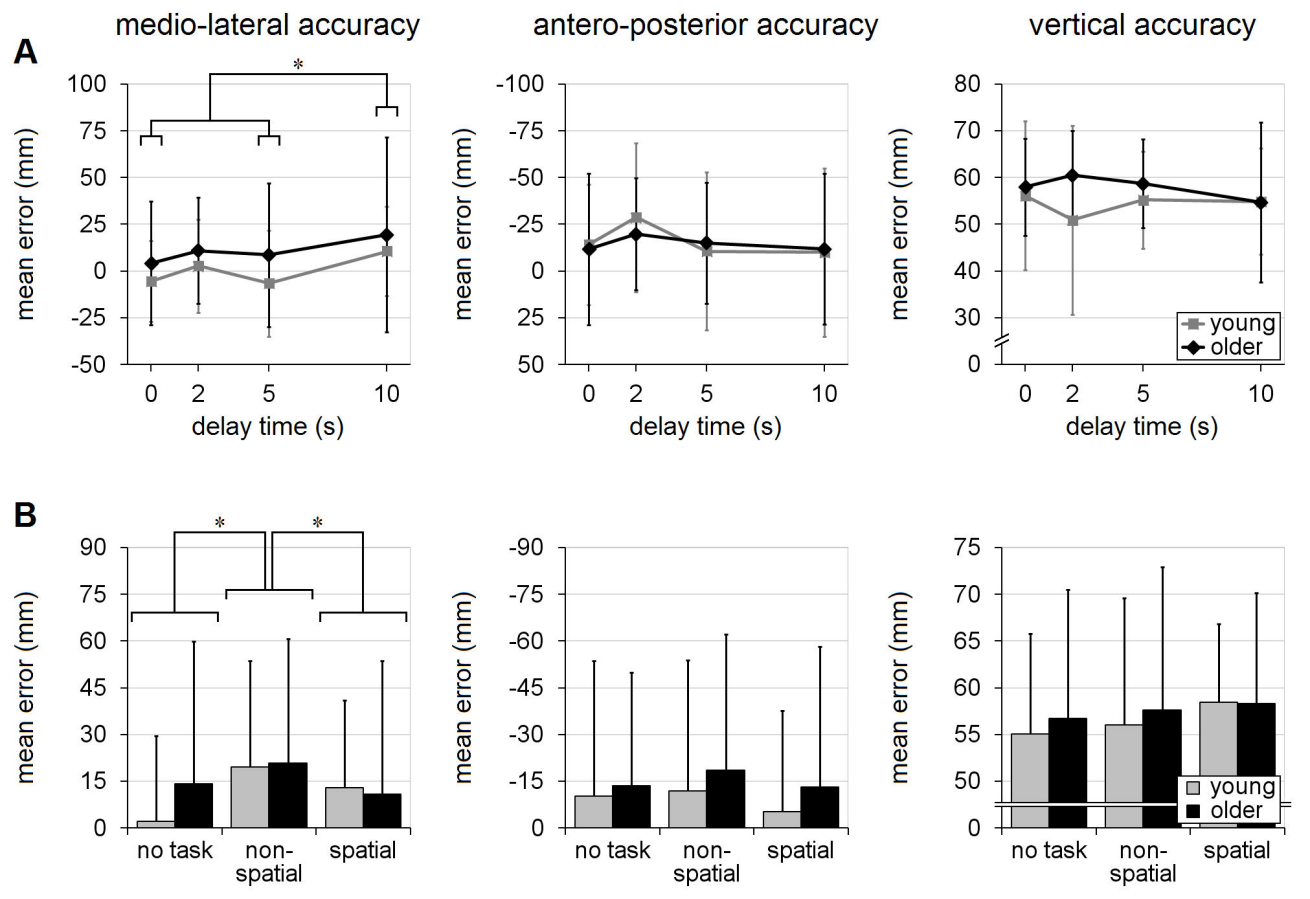
Systematic error. Effects of recall-delay and cognitive-task on reach-to-grasp accuracy (systematic error) for each coordinate axis are shown in (A) and (B), respectively. Means and standard deviations are shown for young adults (gray) and older adults (black). Note the increased mean lateral error during the longest recall-delay trials (DT=10s) in (A), and when performing the non-spatial cognitive task in (B). * indicates a significant difference between means due to recall-delay in (A), or cognitive-task in (B) (α=0.05); whiskers indicate standard deviations. [See Figure 6 caption for note regarding presentation of data in mm].

**Figure 8:**
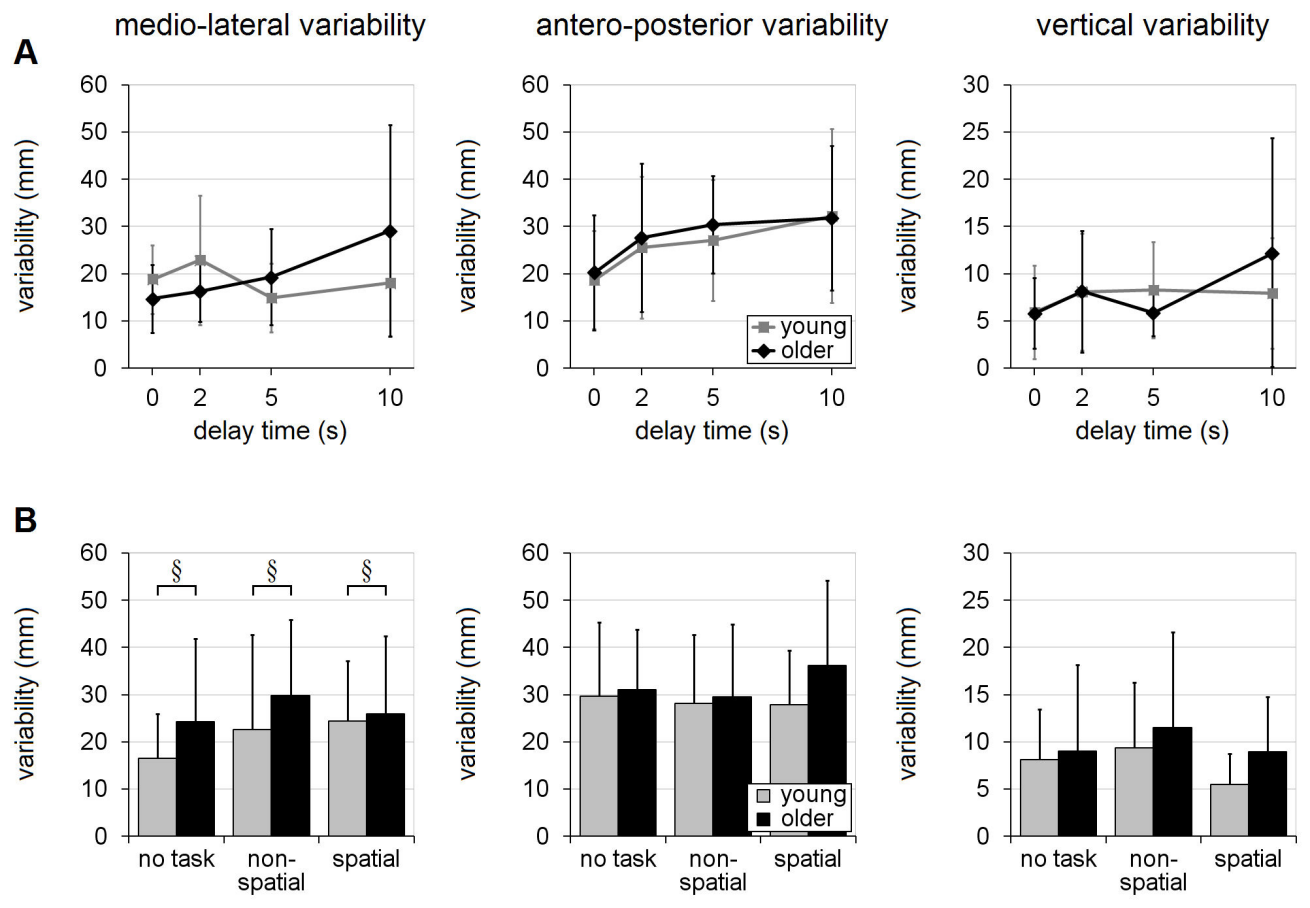
Variable error. Effects of recall-delay and cognitive-task on reach-to-grasp accuracy (variable error) for each coordinate axis are shown in (A) and (B), respectively. Means and standard deviations are shown for young adults (gray) and older adults (black). Note the greater medio-lateral variability by older adults during the longer recall-delay trials (DT=5s/10s) in (B). § indicates a significant difference due to age (main effect); whiskers indicate standard deviations. [See Figure 6 caption for note regarding presentation of data in mm].

The recall-delay analyses, in trials with no secondary cognitive task, indicated that older adults tended to have slower responses than the young adults, across all recall-delay times, in terms of EMG latency, movement-time, time to handhold-contact and time-after-peak-velocity [mean differences of 17-69ms; F’s(1,18)≥7.04, p’s≤0.016; Figure 4A and 5A]. There was, however, no statistical evidence that older adults were more likely to sustain hand-handhold collision (4-5% of trials in each age group; p=0.63) or less likely to achieve a complete grasp (60% of trials vs. 71% in young adults; p=0.51). Analyses of reach trajectory and endpoint error (systematic or variable) also revealed no statistically significant main effects due to age (p's>0.14; Figure 6C, 7A, 8A), although there was an apparent (but not significant, p’s>0.07) tendency for the older adults to transport the hand with a more lateral trajectory; see Figure 6A and 6C. 

**Figure 9:**
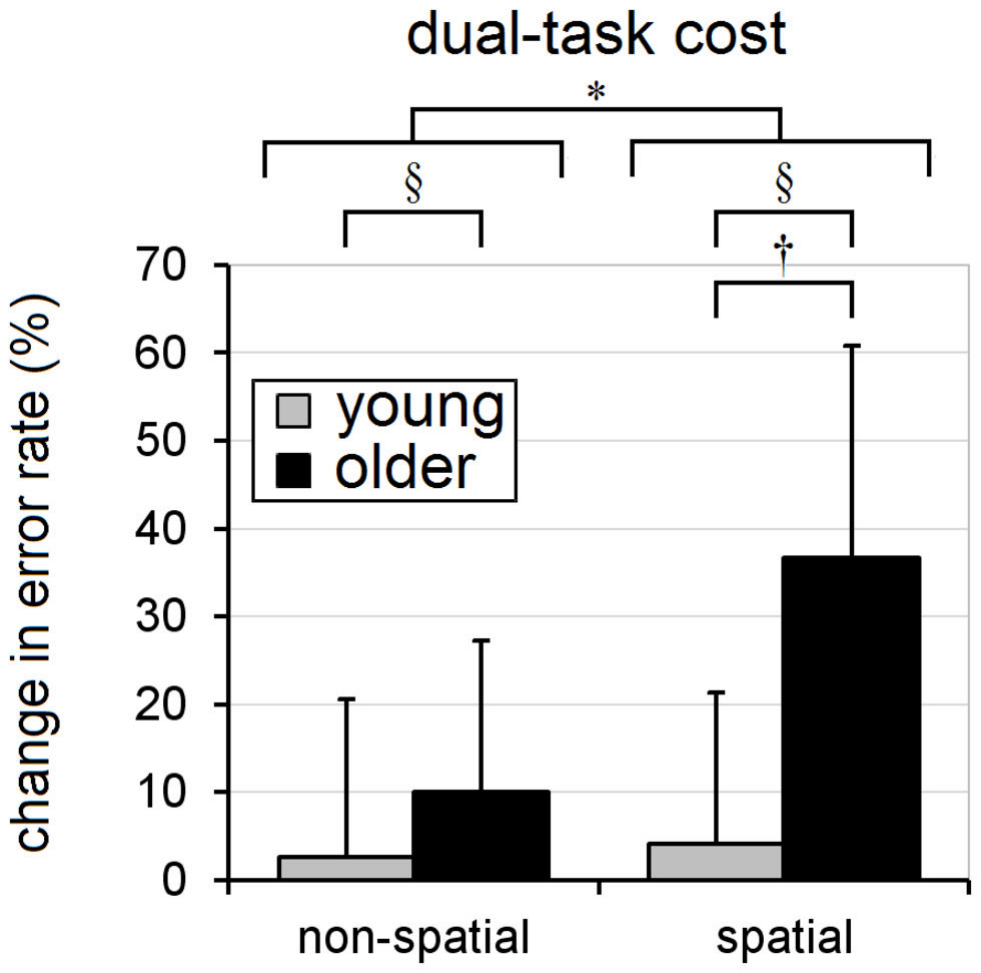
Effect of age on the performance of the two cognitive tasks during the dual-task situation. The y-axis indicates the change in cognitive-task error rate relative to the baseline (single-task) condition. Means and standard deviations are shown for young adults (gray) and older adults (black); whiskers indicate standard deviations. Note the significantly increased error rates in the older adults (for both non-spatial and spatial-tasks), and the amplification of this age-related effect in the spatial-task trials. * indicates a significant main-effect (α=0.05) due to cognitive-task, § indicates a significant main-effect due to age, and † indicates a significant age×task interaction.

 The analyses of cognitive-task effects (i.e. balance-perturbation trials with delay-time of 5s or 10s) further revealed additional main effects due to age, as older adults reached with lower peak transverse-plane velocity [2.19m/s vs. 2.63m/s; F(1,18)=7.09, p=0.016; Figure 5B] and greater medio-lateral endpoint variability [27mm vs. 21mm; F(1,18)=4.64, p=0.045; Figure 8B] than did the younger adults. With regard to cognitive-task performance, the mean rate of incorrect response (across both cognitive tasks) during the balance-perturbation (dual-task) trials was significantly higher, relative to baseline (single-task) trials, in the older adults [error rate increased by 22% vs. 3%; F(1,18)=16.31, p=0.0008; Figure 9 and Table 3].

### Recall-delay and age×delay interaction (trials with no secondary cognitive task)

#### Timing and speed of motion

Recall-delay time (DT) had relatively little effect on the timing and speed of the reactions. There was no significant effect due to recall-delay (or age×delay interaction) on movement-time (p’s≥0.16), and there was only a small but significant main effect on reaction-time and contact-time [~10-25ms slower in no-delay trials; F’s(3,54)≥5.99, p’s≤0.0013]. The slowing in no-delay trials was somewhat more pronounced in the young adults, for contact-time [age×delay interaction: F(3,54)=2.92, p=0.042] but not reaction-time (p=0.44); see Figure 4A. Analysis of peak transverse-plane velocity, time-to-peak-velocity, and time-after-peak-velocity showed no significant differences due to recall-delay main effect nor age×delay interaction (p’s>0.15; Figure 5A).

#### Reach trajectory, accuracy, and variability

Both age groups tended to reach with a slightly more lateral hand trajectory with increasing recall-delay time. This was evident in the deviation from the direct-path trajectory (see Figure 6A for details) and in the analysis of the maximum lateral hand excursion [46mm for DT=10s vs. 29-36mm for DT=0-2s; F(3,54)=6.27, p=0.0010; age×delay interaction, p=0.19; Figure 6C]. Effects on maximum vertical elevation, however, differed in the two age-groups [age×delay interaction: F(3,54)=6.08, p=0.0012]. Specifically, older adults reached to similar maximum vertical clearance for all recall delays (144-158mm), while young adults had a tendency to lift their hand somewhat less high during the shorter-delay trials (107-118mm at DT=0-2s vs. 134-138mm at DT=5-10s); see Figure 6C. In terms of mean endpoint accuracy, recall-delay led to a small increase in lateral error [15.0mm for DT=10s vs. -0.7-7.6mm for DT=0-5s; F(3,54)=4.00, p=0.012] but had no effect on mean accuracy in the other directions (p’s≥0.43); see Figure 7A. The mean-accuracy analyses showed no evidence of an age×delay interaction in any direction (p’s≥0.41), and there were no significant effects due to recall-delay or age×delay interaction in any of the endpoint variability analyses (p’s≥0.11); see Figure 8A. 

#### Prehension

There was a near-significant tendency for full-grasp to occur less frequently with increasing recall-delay [75% of trials for DT=0s and 57-67% for DT=2-10s; F(3,54)=2.62, p=0.060]; however, there was no significant effect on frequency of hand-handhold collision (2-8% of trials for DT=0-10s; p=0.42). Neither of these analyses showed evidence of an age×delay interaction (p’s≥0.45). 

### Cognitive task and age×task interaction (trials with delay-time of 5s or 10s)

#### Cognitive-task performance

Cognitive-task errors occurred more frequently during the spatial-task than the non-spatial task [dual-task error rate increased, relative to the baseline (single-task) rate, by 20% vs. 6%; F(1,18)=6.67, p=0.019], and were more frequent in the older adults in both task conditions. In support of our hypothesis, a significant age×task interaction [F(1,18)=4.44, p=0.0495] further revealed that older adults had a greater tendency to perform worse in the spatial-task than in the non-spatial task during dual-tasking (37% increase in error rate for the spatial-task vs. 10% increase for the non-spatial task within the older adults, vs. 3-4% increase for either task within the young adults; Figure 9). 

#### Timing and speed of motion

Cognitive task had a similar effect on all timing variables in both age-groups [F’s(2,36)≥8.84, p’s≤0.0008; age×task interaction p’s≥0.29]. Specifically, mean reaction-, movement- and contact-time were significantly faster (by ~11-34ms) in the no-task condition; see Figure 4B. Cognitive task had no significant effect on peak velocity (p=0.28); however, performance of the spatial-task led to slightly longer time-after-peak-velocity [by 10ms; F(2,36)=3.88; p=0.030], as well as a small but near-significant tendency for longer time-to-peak-velocity [by 5ms; F(2,36)=3.20; p=0.053], in comparison to no-task trials; see Figure 5B. The analyses of the variables related to peak velocity showed no evidence that the two age groups were affected differently by cognitive-task condition (age×task interaction p’s≥0.38).

#### Reach trajectory, accuracy and variability

Analysis of hand trajectory revealed no significant cognitive-task effect on deviation from the direct-path, maximum lateral deviation or maximum vertical elevation (p’s≥0.16; Figure 6B and 6D). Mean reach error was affected by cognitive-task condition, but only in the medio-lateral direction [F(2,36)=4.70, p=0.015; for other directions, p’s≥0.31], with slightly larger lateral error occurring during non-spatial-task trials (20mm vs. 12mm for spatial-task and 8mm for no-task trials); see Figure 7B. There was no significant main effect due to cognitive-task condition in the variability analyses, in any direction (p’s≥0.12; see Figure 8B), nor was there any evidence of significant age×task interaction for any of the trajectory, accuracy or variability variables (p’s≥0.29).

#### Prehension

There was no significant main effect due to cognitive-task condition (p’s≥0.22) on the frequency of hand-handhold collision (4-8% of trials, across tasks) or frequency of full-grasp (53-62% of trials), nor was there any age×task interaction (p’s≥0.29).

### Evaluation of interactions

As noted in the Methods, there is evidence that analysis of rank-transformed data may not provide a robust test for interactions; therefore, we repeated the analyses on the untransformed data and compared the interaction findings. The analyses of the rank-transformed and untransformed data yielded the same findings, with the following exceptions.  Insignificant age×delay interactions shown by the rank-transformed method became significant in the untransformed recall-delay analyses for: 1) time-to-peak-velocity (p=0.15 vs. 0.022; Figure 5A) and 2) variable medio-lateral error (p=0.11 vs. 0.042; Figure 8A). Conversely, the significant age×delay interaction finding in the recall-delay analysis of contact-time was no longer significant in the untransformed analyses (p=0.042 vs. 0.085; Figure 4A). Given the conflicting findings, these interaction results should be conservatively viewed only as evidence of potential trends.

## Discussion

Like the previously-tested young adults [8], the older adults were generally well able to achieve a functionally-adequate grasp and prevent themselves from falling, despite the challenges imposed by: 1) substantially prolonging the length of time (recall delay) that visuospatial information (VSI) about the handhold location had to be retained in working memory prior to perturbation onset, and 2) performing a concurrent spatial- or non-spatial memory task during the memory-retention interval. This was the case despite a reduction in medio-lateral endpoint accuracy due to recall delay, a slowing of reactions when performing either cognitive task, and a general tendency for the older adults to have slower reach-to-grasp reactions across all experimental conditions. The results did not support our hypothesis that the effect of recall delay on reach accuracy would be exacerbated in the older adults, although there was evidence of an age-related difference in the effect of recall delay on vertical hand elevation. Conversely, our hypotheses regarding the effect of performing the spatial-memory cognitive task were supported, i.e. increased dual-task interference effects in the older adults manifested as impaired cognitive-task performance rather than exacerbated slowing of the reach-to-grasp reactions. 

There is a wealth of dual-task literature supporting the view that specific aspects of balance control require attention and/or other cognitive resources (see reviews [31,32]:). Of particular relevance to the present study are previous findings that visuospatial cognitive tasks were more detrimental to the postural stability of older adults than cognitive tasks that did not have a visuospatial component [18,19]. These studies, however, involved a very undemanding postural task (quiet standing). The present study is, to our knowledge, the first to present evidence that working spatial memory is utilized in the control of perturbation-evoked balance-recovery reactions. Specifically, the observed deterioration in the spatial-memory task performance of the older adults during dual tasking suggests that this cognitive task shared some of the same cognitive resources needed for balance control [33,34], and that the concurrent demands to retain information in spatial working memory exceeded the storage capacity of the older adults [35]. The fact that the dual-tasking affected the cognitive-task performance of the older adults, but did not affect their reach-to-grasp reactions, is consistent with previous evidence suggesting that the CNS will prioritize the balance-recovery task in a situation where stability is challenged [15-17]. 

Interestingly, both spatial and non-spatial cognitive tasks caused similar slowing of the arm reactions, and this was the case in both young and older adults. As we noted in the previous study involving only the young adults [8], we are aware of only one previous study showing that a concurrent cognitive task affects the timing of perturbation-evoked reach-to-grasp reactions [36]. In contrast, other previous studies have found that the cognitive task had relatively little effect on the features of the balance-recovery reactions, whether these reactions involved "feet-in-place" responses [37-40], compensatory stepping [37,41-43], or compensatory reach-to-grasp [44]. In the present study, the cognitive tasks were restricted to the recall-delay interval so as to interfere primarily with accurate retention of the handhold location in spatial working memory; however, it seems unlikely that the spatial task would affect memory retention to a similar degree as the non-spatial task [45]. An alternative explanation is that our subjects continued to attend to each cognitive task for at least some interval of time subsequent to perturbation onset, and that the primary effect of both tasks was due to competing demands for attentional resources needed to retrieve handhold location from visuospatial-memory [46] after perturbation onset. Previous dual-task perturbation-reaction studies have not involved a comparable memory-retrieval requirement, and may have failed to show a dual-task effect on the balance reactions for this reason. 

One of the main effects of recall delay, in both young and older adults, was a reduction in lateral endpoint accuracy. This finding is consistent with results from studies of volitional goal-directed arm-movement studies [47-49], and presumably reflects time-dependent decay in the accuracy of the stored target location [50,51]. However, as we noted in the previous study of young adults [8], the perturbation-evoked responses showed no evidence of the recall-delay-related slowing of movement time, peak velocity and time-after-peak-velocity that has been reported in previous studies of volitional arm movements [49,52-57]. This slowing likely serves to increase the time available to formulate and execute online trajectory corrections [26] based on proprioceptive feedback [58,59]. Presumably, the absence of slowing in the present study reflects the need to react to the perturbation as rapidly as possible, in order to prevent falling; however, the absence of any slowing may reduce the capacity for trajectory corrections and thereby exacerbate the reduction in end-point accuracy arising from memory decay.

A number of age-related differences were observed, across all experimental conditions. Findings that the arm reactions were slower in the older adults are consistent with the age-related slowing that has been observed in previous compensatory grasping studies [7,60,61]. In addition, the present study revealed a tendency for age-related increase in medio-lateral endpoint variability (in the trials with delay times of 5s or 10s), as well as an apparent tendency of the older adults to transport the hand with a more lateral trajectory. The increase in endpoint variability contradicts findings from studies of volitional arm movements, which found that older and young adults were equally able to point accurately to remembered egocentric targets [62,63], but is consistent with age-related deficits in visuospatial memory encoding [10] and/or retention [11,12] that have been reported. 

The less-direct curvilinear hand trajectory in the older adults may reflect age-related declines in the sensorimotor control of arm movement [31] and associated increase in endpoint variability [64-66]. Alternatively, the increased curvature of the trajectory may represent an adaptive strategy to compensate for the uncertainty of the handhold location, by increasing the path-of-travel and hence the likelihood of contacting the handhold [67,68]. Older adults also showed evidence of another possible adaptive strategy, in that they tended to raise the hand high even in the least challenging recall-delay conditions. Age-related tendency to increase elevation of the moving limb to avoid collision with surrounding environmental constraints has also been reported in studies of lower-limb obstacle avoidance during ambulation [69,70]. The present finding may possibly reflect a heightened concern about avoiding handhold collisions, which could be related to increased uncertainty about the handhold location (either due to age-related decline in visuospatial memory [71], or problems with visual perception [70]). 

The general age-related reductions in speed and accuracy noted above would be expected to exacerbate the age-independent reductions in speed and accuracy caused by dual-task interference and recall-delay; however, the older adults in this study were still able to retain and retrieve stored-VSI needed to guide functionally-adequate reach-to-grasp reactions. The lack of overt motor errors such as hand-handhold collision and falls into the safety harness could be partially explained by the easily accessible handhold location and the relatively small perturbation magnitude. Presumably, larger perturbations will require larger stabilizing hand-handhold reaction forces to be generated, and hence may require more accurate reaching in order to ensure that a strong grip is achieved. In addition, larger perturbations will demand more rapid responses, which will reduce the time available to switch attention away from an ongoing cognitive task, retrieve stored VSI about potential handhold locations, encode the motor commands for the reach-to-grasp reaction, and process online multi-sensory feedback to guide/correct the arm trajectory. Further work is needed to determine the influence of recall-delay and dual-tasking on the capacity of young and older adults to respond to larger perturbations. Further work is also needed to determine the degree to which recall-delay and dual-tasking influence the ability to execute effective reach-to-grasp reactions under task conditions that simulate the demands of responding to truly unexpected balance perturbations in the complex environments and situations of daily life. 

Reduced capacity to simultaneously perform cognitive and postural tasks has been suggested as a potential contributor to falls in older individuals with clinical balance impairments [72]. The present results suggest that any cognitive task that draws attention away from the retrieval of visuospatial memory [46,73] and/or the planning and execution of the stabilizing limb movement [32] may delay the initiation and completion of the balance-recovery reaction. When coupled with the general age-related slowing that was observed across all experimental conditions, such delays could well contribute to an increased risk of falling in seniors [60,74]. In particular, older adults with reduced visuospatial memory and attentional capacity [9] may be at greater fall risk if insufficient attentional resources can be allocated to postural control [37], or if they are unable to switch attention promptly from an ongoing motor or cognitive task to the task of recovering balance [14,75]. Further research is needed to determine the effects of memory decay and dual-task interference on reach-to-grasp reactions in older adults with more severe cognitive impairments than the present cohort, and to establish whether interventions aimed to improve visuospatial memory and/or attention capacity can improve balance recovery and thereby reduce risk of falling in various senior populations. 

## Conclusions

Both young and older adults showed a similar reduction in end-point accuracy when forced to guide reach-to-grasp reactions using visuospatial target information that had to be retained in working memory for a substantial length of time (e.g. 10s). Both age groups also demonstrated a similar slowing of reaction onset and completion when required to perform a concurrent spatial or non-spatial memory task prior to perturbation onset. Importantly, however, the older adults also showed a dual-task interference effect (poorer cognitive-task performance) that was specific to the spatial-memory task. This provides new evidence that cognitive resources related to spatial working memory may be utilized in the control of perturbation-evoked balance-recovery reactions. Although the spatial task did not impact reach-to-grasp accuracy, the delays in initiating and completing the reaction that occurred when performing either cognitive task suggest that such dual-task situations in daily life could increase risk of falling in seniors, particularly when combined with the general age-related slowing that was observed across all experimental conditions. Further research is needed to establish whether interventions aimed to improve visuospatial processing and/or attention capacity can reduce risk of falling by improving execution of balance-recovery reactions among senior populations. 
